# Wheat Transcription Factor TaSNAC11-4B Positively Regulates Leaf Senescence through Promoting ROS Production in Transgenic *Arabidopsis*

**DOI:** 10.3390/ijms21207672

**Published:** 2020-10-16

**Authors:** Zenglin Zhang, Chen Liu, Yongfeng Guo

**Affiliations:** Tobacco Research Institute, Chinese Academy of Agricultural Sciences, Qingdao 266101, China; zhangzenglin@caas.cn (Z.Z.); liuchen265936@163.com (C.L.)

**Keywords:** wheat, TaSNAC11-4B, leaf senescence, ROS, stress response

## Abstract

Senescence is the final stage of leaf development which is accompanied by highly coordinated and complicated reprogramming of gene expression. Genetic manipulation of leaf senescence in major crops including wheat has been shown to be able to increase stress tolerance and grain yield. NAC(No apical meristem (NAM), ATAF1/2, and cup-shaped cotyledon (CUC)) transcription factors (TFs) play important roles in regulating gene expression changes during leaf senescence and in response to abiotic stresses. Here, we report the characterization of TaSNAC11-4B (Uniprot: A0A1D5XI64), a wheat NAC family member that acts as a functional homolog of AtNAP, a key regulator of leaf senescence in Arabidopsis. The expression of *TaSNAC11-4B* was up-regulated with the progression of leaf senescence, in response to abscisic acid (ABA) and drought treatments in wheat. Ectopic expression of *TaSNAC11-4B* in Arabidopsis promoted ROS accumulation and significantly accelerated age-dependent as well as drought- and ABA-induced leaf senescence. Results from transcriptional activity assays indicated that the TaSNAC11-4B protein displayed transcriptional activation activities that are dependent on its C terminus. Furthermore, qRT-PCR and dual-Luciferase assay results suggested that TaSNAC11-4B could positively regulate the expression of *AtrbohD* and *AtrbohF*, which encode catalytic subunits of the ROS-producing NADPH oxidase. Further analysis of TaSNAC11-4B in wheat senescence and the potential application of this gene in manipulating leaf senescence with the purpose of yield increase and stress tolerance is discussed.

## 1. Introduction

The leaf is the main photosynthetic organ for the production of energy during plant development and the status of leaves is essential for the efficiency of photosynthesis [1]. As the final stage of leaf development, the occurrence of senescence is accompanied by numerous biochemical and metabolic changes including the degradation of chlorophyll, nucleic acids, and proteins and an increase in relocation or transportation activities [2,3]. Senescence is a genetically programmed process regulated by numerous internal and external factors such as age, nutrient deficiency, stresses, darkness, and pathogen infection [4]. In agriculture, appropriate maintenance of the productive photosynthetic stage is essential to crop yield [2]. Furthermore, the leaf senescence process is critical in the recycling and reallocation of nutrients from vegetative to reproductive tissues to promote seed production and to ensure plants’ survival under harsh environmental conditions [5,6]. The timing of leaf senescence is thus crucial for crop production. Early senescence could reduce plants’ capacity for carbon assimilation and nitrogen uptake, while over-delayed senescence could cause plants to miss the best time for remobilizing nutrients to reproductive organs [5,7].

During leaf senescence, thousands of senescence-associated genes (*SAGs*) are up-regulated, including genes encoding transcriptional factors, signal transduction components, and catabolic enzymes, while photosynthesis-related genes are down-regulated [8,9]. As one of the largest transcriptional factor (TF) families in plants, no apical meristem (NAM), ATAF, and CUC (NAC) TFs participate in diverse aspects of plant development, including leaf senescence [10,11]. For example, AtNAP [12], ORE1 [13], ANAC016 [14], ATAF1 [15], and ANAC072 [16] play positive roles in leaf senescence, while JUB1 [17] and VNI2 [18] showed negative functions during leaf senescence. It is reported that ORE1, together with EIN3 and miR164, formed a regulatory module in ethylene-mediated leaf senescence. As a positive regulator of ethylene signaling, EIN3 directly activated the expression of *ORE1*, while miR164 repressed *ORE1* at the post-transcriptional level [13,19]. Interestingly, previous studies have revealed that *ORE1* was also involved in circadian rhythm-related leaf senescence. PRR9, a core circadian component, positively regulated the expression of *ORE1* by directly binding to its promoter and repressing the expression of miR164, resulting in enhanced expression of *ORE1* and early leaf senescence [20]. JUB1 functions as a negative regulator of leaf senescence in response to H_2_O_2_ level. Overexpression of *JUB1* significantly delayed senescence by reducing H_2_O_2_ accumulation in plants while the *jub1-1* mutant lines showed precocious senescence [17].

NAC TFs were reported to be involved in various biological processes. It was estimated that there are 453 high-confidence NAC protein-coding genes in the wheat genome [21]. NAM1-B1 functions to promote the leaf senescence process, as well the levels of grain proteins and Zn and Fe contents [22]. TaNAC2-5A was reported to be involved in nitrate response and function to increase wheat yield [23]. TaNAC30 [24] and TaNAC21/22 [25] play a negative role in regulating resistance to wheat stripe rust. TaNAC019-A1 negatively regulates the expression of *TaAGPS1* and reduces endosperm starch synthesis [26]. TaNAC2 is involved in multiple stress responses. Overexpression of *TaNAC2* resulted in enhanced tolerances to drought, salt, and freezing stresses in Arabidopsis [27]. TaNAC29 is involved in leaf senescence and responses to salt and drought stresses. Moreover, it has been reported that abscisic acid (ABA) and antioxidant enzyme systems are involved in mediating the function of TaNAC29 [28].

As a NAC protein member, NAP has been reported to be involved in multiple signaling pathways regulating leaf senescence and the role of NAP transcription factors seems to be conserved in different plant species [10,19,29,30,31,32,33]. In Arabidopsis, inducible overexpression of *AtNAP* caused precocious senescence, while plants deficient in AtNAP function displayed significantly delayed leaf senescence phenotypes [12]. Further studies revealed that AtNAP directly regulated the expression of *SAG113*, a gene encoding an ABA and senescence-inducible phosphatase 2C protein. Increasing the expression of *SAG113* rescued the delayed leaf senescence phenotype of the *atnap* mutant [34]. Additionally, it is reported that ABA, AtNAP, and SAG113 together controlled stomatal aperture and water loss in senescing leaves. SAG113 played a negative role in ABA signaling to suppress stomata closure that resulted in rapid water loss in senescing leaves [32]. A recent study revealed that AtNAP was also involved in GA-mediated age-dependent and dark-induced leaf senescence [33]. In rice, OsNAP functions as an important link between ABA and leaf senescence [35]. Expression of *OsNAP* was induced by both age and ABA. Overexpression of *OsNAP* significantly promoted leaf senescence, while knockdown lines of *OsNAP* delayed senescence in rice [35]. Further studies showed that OsNAP positively regulates leaf senescence by directly targeting genes related to chlorophyll degradation and nutrient transport and other genes associated with senescence [35]. Importantly, plants with reduced *OsNAP* expression showed delayed leaf senescence, resulting in an extended grain-filling period and higher grain yield [35,36].

ROS could act as signaling molecules in numerous processes in plants, including disease resistance [37], stress response, and senescence [38]. Under stress conditions, ROS can be generated in apoplasts by the activity of NADPH oxidases [39]. A group of NADPH oxidases/respiratory burst oxidase homologs (Rboh) has been identified in *Arabidopsis thaliana* [40]. There are 10 *Rboh* genes named *AtRboohA* to *AtRbohJ* in the Arabidopsis genome, encoding proteins with different ranges of enzymatic activities and specific functions in plant growth and development [41]. For example, AtRbohB plays a role in after-ripening of Arabidopsis seeds [42]. AtRbohC is involved in root hair tip growth and root hydrotropism [43,44]. AtrbohD and AtrbohF play important roles in ABA-induced ROS production in guard cells [45], as well as in jasmonic acid (JA) signaling [46]. Previous studies revealed that ABA regulated ROS accumulation by affecting transcripts of several ROS-related genes including APX1 and CAT1 in Arabidopsis [47,48]. Additionally, ABA has been shown to induce ROS accumulation and leaf senescence under drought conditions. Reducing ROS production significantly delayed senescence and enhanced drought resistance in tobacco plants [49].

Delay of leaf senescence in wheat as the result of genetic manipulation of senescence-regulating factors has been shown to be able to increase yield significantly, especially under stress conditions [50]. In this study, we characterized the function of TaSNAC11-4B, a wheat NAC transcription factor, in regulating leaf senescence. The expression pattern of *TaSNAC11-4B* was found to be increased during wheat leaf senescence and upon ABA and drought treatments. As a TF, the TaSNAC11-4B protein showed a transcriptional activation activity that was dependent on its C terminus. A complementary assay revealed that TaSNAC11-4B was a functional homolog of AtNAP, a key NAC family regulator of leaf senescence in Arabidopsis [12]. Ectopic overexpression of *TaSNAC11-4B* in Arabidopsis caused leaves to senesce early under natural conditions as well as after drought and ABA treatments. Additionally, TaSNAC11-4B appeared to play a positive role in ROS accumulation by promoting the expression of *AtrbohD and AtrbohF* genes.

## 2. Results

### 2.1. TaSNAC11-4B Expression Is Associated with Leaf Senescence and Can Be Induced by Stress Treatments

**Figure 1:**
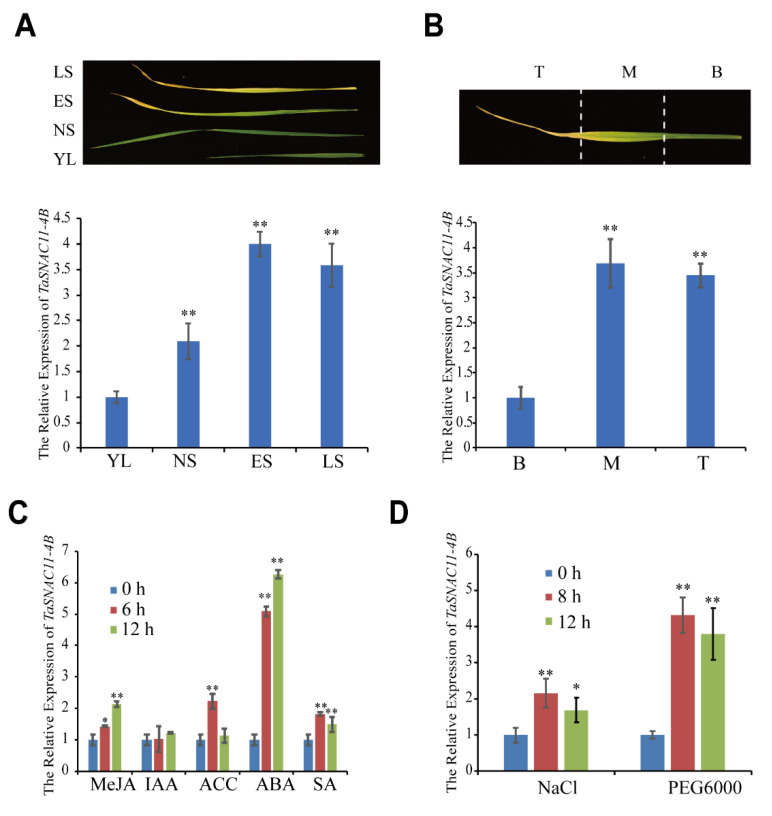
Expression patterns of *TaSNAC11-4B*. (**A**) Expression of *TaSNAC11-4B* in flag leaves at different developmental stages (YL, young leaf; NS, fully expanded leaf without senescence symptoms; ES, early senescent leaf, with <25% leaf area yellowing; LS, late senescent leaf, with >50% leaf area yellowing). (**B**) Expression of *TaSNAC11-4B* in different parts of one single flag leaf (B, base; M, middle; T, tip). (**C**) Expression patterns of *TaSNAC11-4B* in wheat leaves under different plant hormone treatments as indicated. (**D**) Expression patterns of *TaSNAC11-4B* in wheat leaves after NaCl, PEG6000 treatments. The 2*^−^^ΔΔCt^* method was used in qRT-PCR analysis. *TaGAPDH* was used as an internal control. Asterisks indicate statistically significant differences (* *p* < 0.05; ** *p* < 0.01) using a Student’s *t*-test. Three independent experiments were performed.

Via a BLAST search against the wheat protein database in the Ensembl Plants website (http://plants.ensembl.org/index.html) using the OsNAP protein sequence as a query, we obtained a wheat NAC transcriptional factor homologous to the senescence regulator NAP (Figure S1), previously named TaSNAC11-4B [51]. To investigate the potential roles of TaSNAC11-4B in wheat leaf senescence, we performed qRT-PCR to analyze its expression profiles at different development stages in flag leaves. The results indicated a significant increase in *TaSNAC11-4B* expression at early stages of leaf senescence (Figure 1A). Within a single flag leaf undergoing senescence, the expression of *TaSNAC11-4B* increased from the younger base to the yellowing tip (Figure 1B). Given that NAP TFs as well as a number of other NAC TF family members have been shown to be involved in stress responses [27,35,52,53], we examined the expression of *TaSNAC11-4B* in detached leaves after treatment with phytohormones ABA, MeJA, 1-aminocyclopropane-1-carboxylic acid (ACC), salicylic acid (SA), indole-3-aceticacid (IAA), and stressors including NaCl and drought (PEG6000 mimic). Expression of the *TaSNAC11-4B* gene was induced by all the senescence-promoting phytohormones, ABA, JA, ethylene, and SA, with an increase of more than sixfold 12 h after ABA treatment (Figure 1C). In addition, *TaSNAC11-4B* expression was significantly induced by both NaCl and PEG6000 treatments. Based on the results of expression analyses, we propose that TaSNAC11-4B likely plays a role in age-dependent and stress-induced leaf senescence in wheat.

### 2.2. TaSNAC11-4B Is a Functional Homolog of AtNAP

**Figure 2:**
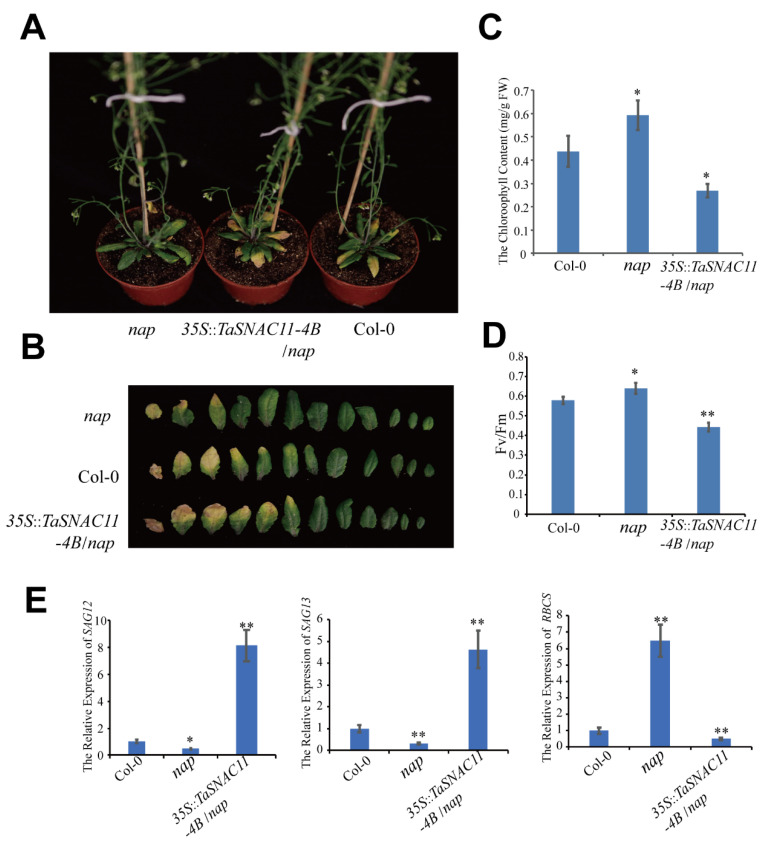
Complementation of Arabidopsis *nap* null plants with *TaSNAC11-4B*. (**A**) Phenotypes of 5-week-old whole plants of Col-0, *nap* null mutant, and a complementation line. (**B**) Phenotypes of individual rosette leaves of Col-0, *nap* null mutant, and the complementation line. (**C**,**D**) Chlorophyll content and Fv/Fm ratio of the sixth leaf from plants of different genotypes. (**E**) Expression of senescence marker genes in Col-0, *nap* mutant, and complementation line. Asterisks indicate statistically significant differences (* *p* < 0.05; ** *p* < 0.01) using a Student’s *t*-test. Three independent experiments were performed.

To test whether *TaSNAC11-4B* is a functional homolog of the senescence-regulating AtNAP, a complementary assay was carried out. Transgenic lines overexpressing *TaSNAC11-4B* driven by the *CaMV35S* promoter were obtained in the *atnap* mutant background of Arabidopsis. As previously reported [12], leaf senescence of the *atnap* plants was significantly delayed in comparison with WT Col-0 (Figure 2). The delayed senescence phenotype was reversed when *TaSNAC11-4B* was overexpressed in the *atnap* plants (Figure 2). Phenotypical analysis of 5-week-old plants showed that when five to six leaves of *35S::TaSNAC11-4B/atnap* and Col-0 plants showed visible yellowing, only three leaves of *atnap* plants showed yellowing (Figure 2A,B). Compared with Col-0, *atnap* mutant leaves had higher, while the *35S::TaSNAC11-4B/atnap* leaves showed lower chlorophyll content and Fv/Fm rates (Figure 2C,D). The other independent lines displayed similar results (Figure S4) Consistent with the visible phenotypes, transcript levels of the senescence marker genes *SAG12* and *SAG1*3 were higher, while expression of the photosynthesis gene *RBCS* was lower in *35S::TaSNAC11-4B/atnap* and Col-0 plants compared with *atnap* plants (Figure 2E). Ectopic expression *TaSNAC11-4B* could restore the Arabidopsis *atnap* mutant back to WT, indicating that the wheat TF TaSNAC11-4B is a functional homolog of AtNAP in regulating leaf senescence.

### 2.3. TaSNAC11-4B Plays a Positive Role in Regulating Leaf Senescence

**Figure 3:**
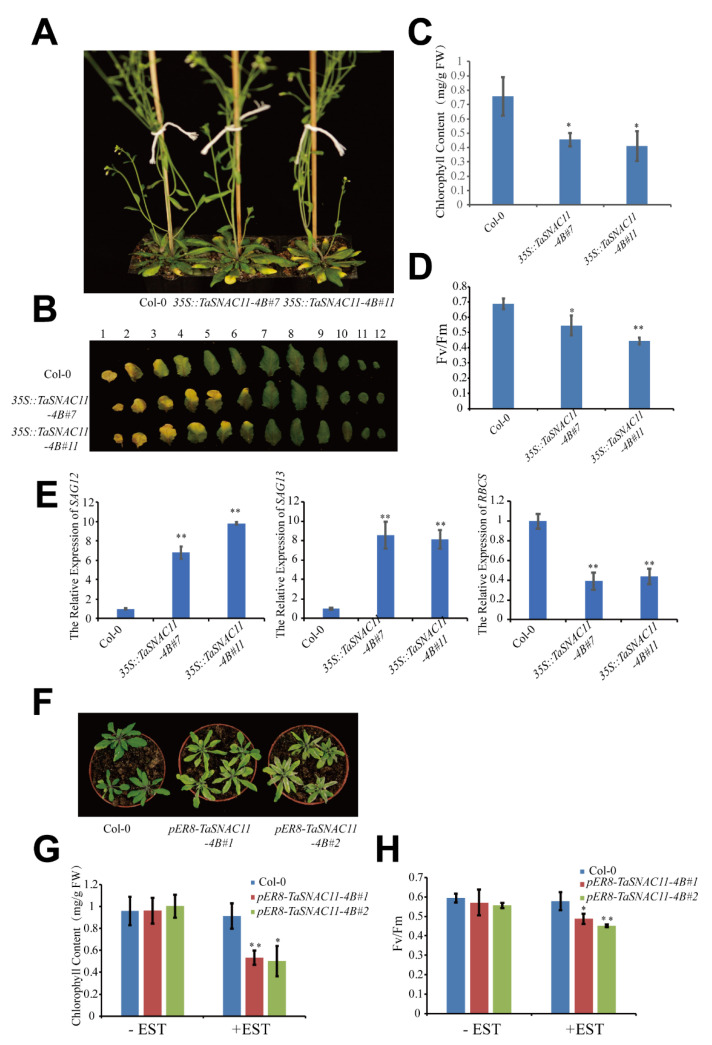
*TaSNAC11-4B* positively regulates leaf senescence in transgenic Arabidopsis. (**A**) Six-week-old plant phenotypes of transgenic Arabidopsis plants overexpressing *TaSNAC11-4B*. (**B**) Detached leaf phenotypes of Col-0 and *TaSNAC11-4B* overexpression lines. (**C**,**D**) Chlorophyll content and Fv/Fm ratio of the sixth leaf in plants with different genotypes as indicated. (**E**) Expression of *SAG12*, *SAG13*, and *RBCS* in Col-0 and *TaSNAC11-4B* overexpressing transgenic Arabidopsis. (**F**) β-estradiol (EST)-inducible overexpression of TaSNAC11-4B causes precocious senescence. (**G**,**H**) Chlorophyll content and Fv/Fm ratio of leaves from different plants that were treated with or without EST. Asterisks indicate statistically significant differences (* *p* < 0.05; ** *p* < 0.01) using a Student’s *t*-test. Three independent experiments were performed.

To further study the function of *TaSNAC11-4B* in regulating leaf senescence, we overexpressed *TaSNAC11-4B* in WT Col-0. Two independent transgenic lines (*35S*::*TaSNAC11-4B*#7, *35S*::*TaSNAC11-4B*#11) were obtained and the expression of *TaSNAC11-4B* was confirmed by RT-PCR (Figure S2). For 6-week-old plants, the 1st to 3rd leaves of Col-0 showed visible yellowing when yellowing was observed on the 1st to 6th leaves of transgenic plants harboring *35S*::*TaSNAC11-4B*#7 or *35S*::*TaSNAC11-4B*#11 (Figure 3A,B). The difference in senescence phenotype was also confirmed by chlorophyll content and Fv/Fm ratio, with leaves of *35S*::*TaSNAC11-4B*#7 and *35S*::*TaSNAC11-4B*#11 having lower chlorophyll content and Fv/Fm values compared with that of Col-0 (Figure 3C,D). Furthermore, the expression levels of *SAG12* and *SAG13* were significantly higher, while *RBCS* expression was reduced in *35S*::*TaSNAC11-4B*#7 and *35S*::*TaSNAC11-4B*#11 plants compared with Col-0 (Figure 3E), indicating that enhanced expression of *TaSNAC11-4B* in Arabidopsis can accelerate the process of leaf senescence.

To further confirm the results of the overexpression study, we used a chemical-inducible overexpression strategy in which *pER8-TaSNAC11-4B* was generated to transform Arabidopsis to obtain β-estradiol (EST)-inducible gain-of-function transgenic lines. Treatments with 10 μM EST of plants of two independent lines, pER8-*TaSNAC11-4B*#1 and pER8-*TaSNAC11-4B*#2, but not of Col-0, caused induced expression of *TaSNAC11-4B* (Figure S3). Precocious leaf yellowing and a significant reduction in chlorophyll content and Fv/Fm ratio were observed in plants with induced expression of *TaSNAC11-4B* (Figure 3F–H).

### 2.4. TaSNAC11-4B Functions to Promote Drought-Induced Leaf Senescence

**Figure 4:**
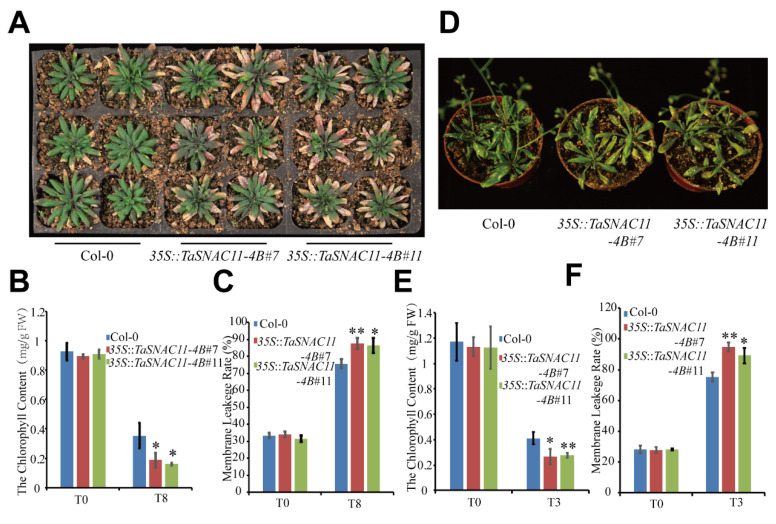
Overexpression of *TaSNAC11-4B* in Arabidopsis accelerates drought-induced leaf senescence. (**A**) Phenotype analysis of 32-day-old plants under water deficit conditions. Six-week-old plants grown in soil were subjected to a water shortage treatment for 10 days and photos were taken one day after the plants were re-watered. (**B**) Chlorophyll content of different plants before and after 8 days’ water deficit treatment. (**C**) Membrane leakage rate of Col-0 and *TaSNAC11-4B* overexpression plants before and after 8 days’ water deficit treatment. (**D**) Phenotypic characteristics of 3-week-old Col-0 and *TaSNAC11-4B* overexpression plants under mannitol treatments. Col-0 and *TaSNAC11-4B* overexpression lines grown in soil were irrigated with 200 mM mannitol for 5 days. (**E**) Chlorophyll content of Col-0 and *TaSNAC11-4B* overexpression plants before and after 3 days of mannitol treatment. (**F**) Membrane leakage rate of Col-0 and *TaSNAC11-4B* overexpression plants before and after 3 days of mannitol treatment. Asterisks indicate statistically significant differences (* *p* < 0.05; ** *p* < 0.01) using a Student’s *t*-test. Three independent experiments were performed.

Considering that the expression of *TaSNAC11-4B* was up-regulated by ABA and drought, we investigated the senescence phenotype of *TaSNAC11-4B* overexpression lines under drought conditions. Six-week-old plants were subjected to water deficit treatments for 10 days and significantly more wilting and yellowing were observed in plants of *35S*::*TaSNAC11-4B*#7 and *35S*::*TaSNAC11-4B*#11 than that of Col-0 (Figure 4A). The results of chlorophyll content analysis were consistent with the visible phenotype (Figure 4B). Stress conditions often compromise the integration of plant cell membranes, leading to ion leakage [54,55]. After water deficit treatments, leaves of *TaSNAC11-4B* overexpression lines displayed significantly higher membrane leakage rates than that of Col-0 (Figure 4C), demonstrating that expression of *TaSNAC11-4B* indeed led to higher sensitivity to drought stress.

In another treatment mimicking drought stress, 200 mM mannitol was applied to 3-week-old plants and 5 days after treatment, plants from both *TaSNAC11-4B* overexpression lines displayed more serious leaf yellowing compared with Col-0 (Figure 4D). Results from chlorophyll content and membrane leakage rate measurements were consistent with the visible phenotype (Figure 4E,F).

### 2.5. TaSNAC11-4B Is Involved in ABA-Induced Leaf Senescence

**Figure 5:**
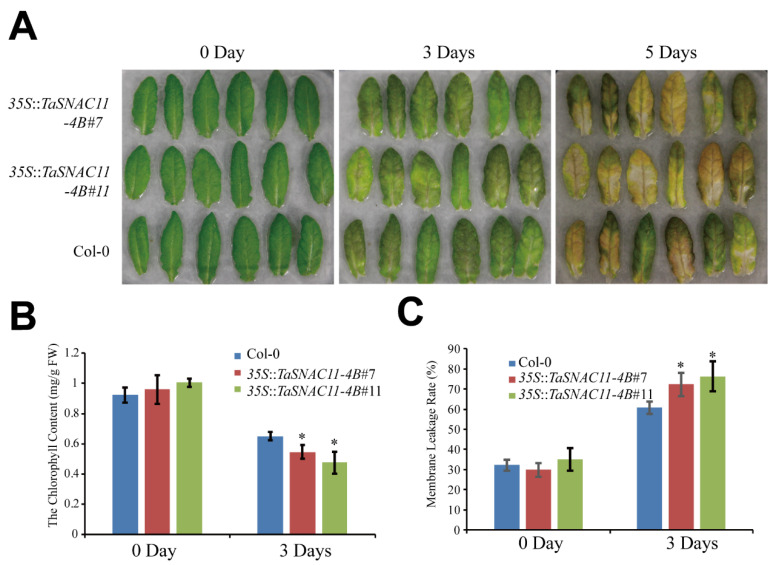
*TaSNAC11-4B* enhances ABA-induced leaf senescence (**A**) Detached leaf phenotypes of Col-0 and *TaSNAC11-4B* overexpression plants after treatment with abscisic acid (ABA). (**B**,**C**) Chlorophyll content and Fv/Fm ratio of detached leaves from Col-0 and *TaSNAC11-4B* overexpression plants treated with ABA for 3 days. Asterisks indicate statistically significant differences (* *p* < 0.05) using a Student’s *t*-test. Three independent experiments were performed.

ABA plays essential roles in a plant’s response to stresses and in leaf senescence [56]. Given that *TaSNAC11-4* expression was enhanced by exogenous application of ABA, we investigated phenotype changes of detached leaves after treatment with 5 μM ABA for 5 days. Detached leaves of *35S*::*TaSNAC11-4B*#7 and *35S*::*TaSNAC11-4B*#11 displayed accelerated senescence compared with those of Col-0 (Figure 5A) and the results of chlorophyll content and membrane leakage rate were in accordance with the visible phenotype (Figure 5B,C), indicating that TaSNAC11-4B functions to enhance ABA-mediated leaf senescence.

### 2.6. Transcriptional Activity of TaSNAC11-4B

**Figure 6:**
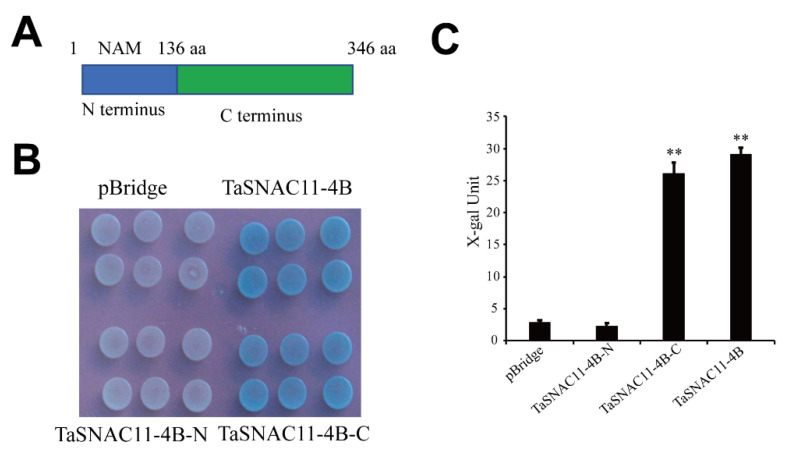
Transcriptional activity determination in yeast cells. (**A**) TaSNAC11-4B protein domain structure. (**B**) Yeast cells transformed with pBridge-TaSNAC11-4B-N, -TaSNAC11-4B-C, full-length TaSNAC11-4B, and empty vector, grown on SD-Trp-Ade media. (**C**) X-gal activity of yeast cells harboring different constructs as indicated. Asterisks indicate statistically significant differences (** *p* < 0.01) using a Student’s *t*-test. Three independent experiments were performed.

We tested the transcriptional activity of the TaSNAC11-4B protein in yeast cells. The full-length coding sequence (CDS )of *TaSNAC11-4B*, N-terminal partial protein coding region *TaSNAC11-4B-N*, or C-terminal partial protein coding region *TaSNAC11-4B-C* (Figure 6A) was fused to the yeast GAL4 DNA-binding domain in the pBridge vector, which was used in transforming yeast strain AH109. The yeast transformants grown on the synthetic defined medium (SD)/Trp for 3 days were transferred to SD/Trp medium supplied with α-gal to assess X-galactosidase activities by liquid assay using chlorophenolred-β-D-galactopyranoside(CPRG) as substrate. After 3 days’ incubation on the α-gal plates, the yeast cells harboring full-length *TaSNAC11-4B* or *TaSNAC11-4B-C* displayed a blue color while those harboring *TaSNAC11-4B-N* or the pBridge empty vector displayed a white color (Figure 6B). The X-gal activity values from full-length *TaSNAC11-4B* and *TaSNAC11-4B-C* were 29.09 ± 1.01 and 26.12 ± 1.74, respectively. On the other hand, the X-gal activity value for *TaSNAC11-4B-N* was 2.32 ± 0.37 and that for the empty vector was 2.89 ± 0.31 (Figure 6C). Together, these data indicated that *TaSNAC11-4B* displayed transcriptional activation activities dependent on the C-terminal region that contains the transcription regulatory domain [57].

### 2.7. TaSNAC11-4B Enhances ROS Accumulation by Activating Atrboh Genes

**Figure 7:**
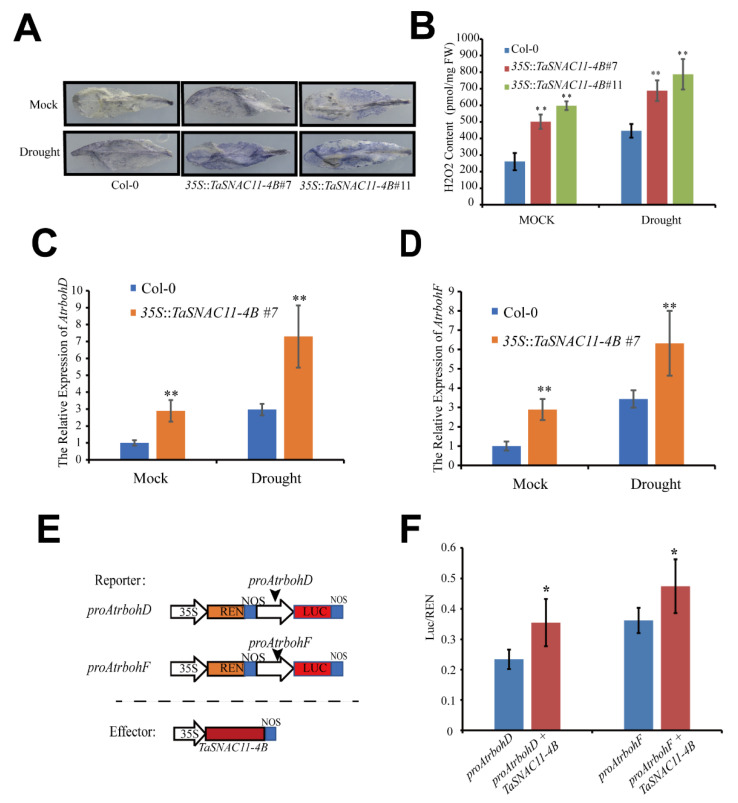
*TaSNAC11-4B* positively regulates ROS accumulation. (**A**) NBT staining in Col-0 and *TaSNAC11-4B* overexpression plants. (**B**) Quantification of endogenous H_2_O_2_ content in Col-0 and *TaSNAC11-4B* overexpression plants under normal and drought stress conditions. (**C**,**D**) Expression analysis of *AtrbohD* and *AtrbohF* genes in Col-0 and *TaSNAC11-4B* overexpression plants. (**E**) Schematic diagram of the reporter and effector constructs used in the dual luciferase assay. (**F**) TaSNAC11-4B increased the expression of *Atrboh* genes. Promoter regions of *AtrbohD* and *AtrbohF* were fused with firefly Luciferase and used in co-transformation with the *35S*:: *TaSNAC11-4B* vector into *Nicotiana benthamiana* leaves. Renilla Luciferase activity serves as an internal control. Asterisks indicate statistically significant differences (* *p* < 0.05; ** *p* < 0.01) using a Student’s *t*-test. Three independent experiments were performed.

ROS are important signaling molecules in plants in response to a variety of stresses, as well as during senescence [58]. To investigate the role of TaSNAC11-4B in regulating ROS accumulation, the sixth leaves of Arabidopsis plants were detached and immersed in Nitro-blue tetrazolium (NBT) staining solution to visualize H_2_O_2_ levels. The results showed that leaves of *TaSNAC11-4B* overexpression lines displayed enhanced color compared with that of Col-0 (Figure 7A). The results from H_2_O_2_ quantification were consistent with the chemical staining data (Figure 7B), indicating that expression of *TaSNAC11-4B* promoted ROS accumulation in Arabidopsis leaves. The NADPH oxidase-mediated pathway has been shown to be the most prominent pathway in ROS production [59,60,61]. In Arabidopsis, two of the NADPH oxidase-encoding genes, *Atrboh D* and *Atrboh F*, have been shown to play key roles in ABA-mediated ROS production [45]. The results in this study demonstrated that TaSNAC11-4B functions in accelerating ABA- and stress-induced leaf senescence. Therefore, we tested whether *AtrbohD* and *Atrboh F* are related to the role of TaSNAC11-4B in promoting ROS production and leaf senescence. Interestingly, the qRT-PCR results showed that the transcripts of *AtrbohD* and *AtrbohF* were significantly up-regulated in *TaSNAC11-4B* overexpression lines compared to Col-0 under both normal and drought conditions (Figure 7C,D, Figure S5). Furthermore, we employed a dual-Luciferase assay in leaves of *Nicotiana benthamiana* to investigate the regulation of these *Atrboh* genes by *TaSNAC11-4B*. In the reporter constructs, the *LUC* gene was under the control of the *AtrbohD* or *AtrbohF* promoter. In the same construct, Renilla acts as a native control driven by the *35S* promoter (Figure 7E). In the effector construct, *TaSNAC11-4B* was driven by the *35S* promoter. As shown in Figure 7F, the LUC/REN ratio, as an indicator of direct transcription regulation, was significantly higher in reporter constructs co-transformed with the effector construct compared with the empty vector, suggesting that the TaSNAC11-4B transcription factor could directly regulate expression of the *Atrboh* genes.

## 3. Discussion

As a complicated but orderly controlled biological process, leaf senescence is regulated by various internal and external factors including age, phytohormones, and environmental cues [9]. Appropriate timing of senescence is important for plant development and productivity. In particular, leaf senescence in crop species has a great impact on yield and quality due to its crucial roles in photosynthetic activities and nutrient recycling [62]. Studies on the molecular mechanisms underlying leaf senescence thus have both biological and agricultural significance [63].

As the largest plant specific TF family, NACs play essential roles in a variety of stress response and developmental processes, including leaf senescence [11]. A number of NAC members have been well characterized for their roles in regulating leaf senescence, including AtNAP [12], ORS1 [64], ORE1 [65], ANAC016 [14], JUB1 [17], and VNI2 [18]. Among these NAC TFs, ANAC046 [66], ANAC072 [16], and AtNAP [12] are positive regulators, while JUB1 [17] is a negative regulator of leaf senescence. In wheat, a NAC transcription factor (encoded by *NAM-B1*) was reported to play a role as a positive regulator in leaf senescence and nutrient remobilization in ancestral wild wheat but this gene seems to be non-functional in modern wheat varieties [22]. In this study, we characterized the function of TaSNAC11-4B in leaf senescence. Firstly, we found that the transcripts of *TaSNAC11-4B* increased in aging leaves (Figure 1). A complementary assay revealed that TaSNAC11-4B can rescue the delayed senescence phenotype caused by a loss of function of *AtNAP* in Arabidopsis, providing additional evidence supporting the idea that the function of NAP in senescence is conserved in dicotyledon and monocotyledon plants (Figure 2). Next, the constitutive and inducible overexpression of *TaSNAC11-4B* caused early senescence of Arabidopsis leaves under normal or drought stress conditions, indicating that TaSNAC11-4B plays a positive role in age-dependent and stress-induced leaf senescence.

In addition to senescence, NAC TFs play important roles in plants’ response to abiotic stresses, including drought and salt stress [36,52]. AtNAP has been reported to function as a negative regulator of salt tolerance. Compared to wild type, loss-of-function mutants of *AtNAP* were more tolerant to salt stress, whereas *AtNAP*-overexpressing transgenic plants were more sensitive to salt stress [53]. A number of wheat NAC TFs have been characterized for their function in stress response. TaNAC29 has been shown to play a regulatory role in salt and drought stress response, as well leaf senescence, via suppressing the expression of *SAG113*, which is involved in drought stress and ABA response [28,32]. Transgenic plants overexpressing *TaNAC69* caused improved drought tolerance in wheat [67]. Moreover, TaNAC2 and TaNAC67 conferred tolerance to multiple abiotic stresses in transgenic plants [27,68]. In this study, we found that *TaSNAC11-4B* expression was up-regulated by drought stress (Figure 1D). Overexpression of *TaSNAC11-4B* in Arabidopsis enhanced the drought-induced senescence process (Figure 4). When suffering from drought stress, plants often start senescence early in order to increase the chance of survival because nutrient remobilization from senescing leaves could support reproductive growth or development of young tissues. *TaSNAC11-4B* serves as a positive regulator in ROS production under drought stress, leading to programmed cell death (PCD) which accelerated the senescence process. The function of *TaSNAC11-4B* in drought-induced senescence made plants more adaptive to unfavorable growth conditions.

As an essential plant hormone, ABA is involved in a variety of plant responses to environmental stresses [69]. The role of ABA in leaf senescence has also been well studied [70]. Previous studies have revealed that several ABA-inducible *SAG*s are involved in the regulation of leaf senescence, including the NAC TFs AtNAP [12,34] and VNI2 [18]. AtNAP has been shown to regulate expression of ABA biosynthetic genes including *NCED2*, *ABA3*, and *AAO3.* AtNAP promoted the expression of *AAO3* by directly binding to its promoter and increased *AAO3* expression in Arabidopsis *atnap* mutants could inhibit the delayed leaf senescence phenotype [71]. AtNAP functions by promoting leaf senescence via up-regulating ABA biosynthetic genes, thus enhancing ABA accumulation [33,71]. In this study, *TaSNAC11-4B* displayed a significant expression increase under ABA treatments (Figure 1C). Overexpression of *TaSNAC11-4B* accelerated ABA-induced senescence, which is likely the reason of that *TaSNAC11-4B* plays a positive role in regulating drought-induced senescence.

Furthermore, a number of NAC family members have been reported to be involved in ROS accumulation. For example, JUB1 acts as a negative regulator in leaf senescence through suppressing intracellular H_2_O_2_ production [17]. NTL4, a drought-responsive NAC TF, mediates drought-induced leaf senescence by inducing *AtRbohC* and *AtRbohE* and enhancing ROS accumulation [72]. ROS are important signaling molecules involved in both stress response and leaf senescence [73]. In this study, the results of both NBT chemical staining and H_2_O_2_ quantification indicated that *TaSNAC11-4B* positively regulated ROS accumulation (Figure 7A,B). Further study revealed that *TaSNAC11-4B* positively regulated the expression of NADPH oxidase subunit-encoding genes, including *AtrbohD* and *AtrbohF*. Previous studies have demonstrated that the *Atrboh* genes were involved in ABA-mediated stomatal closing, defense response, and plant developmental processes [45,74,75]. Interestingly, we also observed that *TaSNAC11-4B* could enhance ABA-induced leaf senescence. We thus speculate that *TaSNAC11-4B* mediates age-dependent and drought- and ABA-induced leaf senescence partially via regulating ROS production via regulating ROS biosynthetic genes including *AtrbohD* and *AtrbohF*. More and more evidence has demonstrated that regulators of leaf senescence are usually related to crop yield and stress response. The Loss of function of NAM-B1, a wheat NAC protein, resulted in delayed leaf senescence and a significant reduction in grain zinc, iron, and protein content [22]. OsNAP plays positive roles in regulating senescence, and reduced *OsNAP* expression led to an extended grain filling period and higher grain yield [35]. Overexpression of *TaNAC29* significantly increased tolerance to salt and drought stresses and delayed leaf senescence in Arabidopsis [28]. Our data indicated that *TaSNAC11-4B* was involved in leaf senescence and drought response. Further study is needed to understand the role of *TaSNAC11-4B* in yield improvement and stress tolerance.

## 4. Materials and Methods

### 4.1. Plant Materials and Growth Conditions

Bread wheat Chinese Spring (*Triticum aestivum L*. cv. Chinese Spring) was used for *TaSNAC11-4B* gene cloning. Arabidopsis ecotype Col-0 was used for transgenic studies. Different stages of wheat grown in a greenhouse were used for gene expression detection (YL, young leaves, were from plants grown for 40 days; NS, fully expanded leaves without senescence symptoms, were from plants grown for 60 days; ES, early senescent leaves, with <25% leaf area yellowing, were from plants grown for 80 days; LS, late senescent leaves, with >50% leaf area yellowing, were from plants grown for 110 days). Arabidopsis seeds were surface sterilized with 70% (*v*/*v*) ethanol for 5 min, then washed three times with distilled water for 5 min each time. Seeds were transferred to 0.5× Murashige and Skoog(MS) medium plates and stratified at 4 °C for 3 days in darkness before being moved to continuous light conditions at 22 °C for germination and growth.

### 4.2. Phytohormone and Stress Treatments

For stress treatments of wheat, detached fully expanded flag leaves were immersed in 150 mM NaCl or 20% (*v*/*v*) polyethylene glycol 6000 (PEG6000) for the indicated time. For plant hormone treatments, detached fully expanded flag leaves were immersed in 10 μM ABA, 50 μM MeJA, 100 μM SA, 50 μM IAA, or 10 μM ACC solution for the indicated time. Samples were collected for RNA extraction or stored at −80 °C for future analysis.

For Arabidopsis, mannitol treatments were conducted by irrigating 3-week-old plants with 200 mM mannitol. For water deficit assays, irrigation was withheld for 10 days. In ABA treatments, the sixth leaves were detached from 4-week-old plants and incubated on filter papers soaked with 5 μM ABA.

### 4.3. Construct Generation and Arabidopsis Transformation

The full-length coding region of *TaSNAC11-4B* was amplified from bread wheat cDNA using primers *TaSNAC11-4B-OEF* and *TaSNAC11-4B-OER* (Table S1). The PCR products were cloned into pDonor-zero to form *pDonor-TaSNAC11-4B*. Then the CDS region of *TaSNAC11-4B* was shuttled to a pEarlyGate202 or pER8 vector by gateway method, forming *35S*::*TaSNAC11-4B* and pER8-*TaSNAC11-4B*, respectively. The full-length protein, N-terminal NAM domain (amino acids 1–136) and the C-terminal domain (amino acids 137–346) encoding DNA fragments, were PCR amplified and sub-cloned into the pBridge vector at the EcoRI and BamHI restriction enzyme sites to form *TaSNAC11-4B*-, *TaSNAC11-4B-N*-, and *TaSNAC11-4B-C*-pBridge constructs. *Agrobacterium* strain GV3101 harboring the stated constructs was transformed into Arabidopsis using the floral dip method. T0 seedlings grown on soil for two weeks were sprayed with 0.1% Glufosinate ammonium(BASTA) solution to select the potential positive transgenic plants which were further confirmed by qRT-PCR. T3 plants of positive lines were used for phenotyping and physiological studies.

### 4.4. β-Estradiol (EST) Treatments

Twenty-one-day-old Arabidopsis plants grown under continuous light were sprayed with 10 μM EST once a day for 2 days. The treated plants were incubated for three additional days before leaves were harvested for RNA extraction, chlorophyll content determination, and Fv/Fm ratio analysis.

### 4.5. Chlorophyll, Fv/Fm, and Electrolyte (Ion) Leakage Rate Measurement

Chlorophyll was extracted from leaves with 100% ethanol. Chlorophyll content was determined by measuring absorbance at 646.6 nm and 663.6 nm. Results were calculated as previously described [76]. The ratio of variable to maximal fluorescence (Fv/Fm) was measured by a chlorophyll fluorometer (OPTI-SCIENCES, NH, USA) according to the manufacturer’s instructions. Membrane ion leakage was determined using a bench-top conductivity meter (Leici, DDS-11A, INESA Instrument, Shanghai, China). Briefly, detached leaves were washed three times with deionized water followed by shaking in deionized water for 1 h. Then the conductivity was measured as initial data, and samples were then boiled and conductivity was measured again as final data when the solution cooled down to room temperature. Total electrolyte leakage was determined by dividing the initial data with the final data. The experiments were repeated three times.

### 4.6. Quantitative RT-PCR Assay

Total RNA was extracted from leaves using TRIZOL reagent (Invitrogen, CA, USA) according to the manufacturer’s instructions. The cDNA was synthesized using total RNA then treated with DNase and subjected to reverse transcription using the Prime Script RT reagent kit (TAKARA, Shiga, Japan). The qRT-PCR was performed on an Applied Biosystems 7500 Fast Real-Time PCR System with three technical and three biological replicates. Actin2 and TaGAPDH were used as internal controls in Arabidopsis and wheat, respectively. The data analysis was performed according to the 2*^−ΔΔCt^* method [77].

### 4.7. TF Transcriptional Activity

Sequencing-confirmed constructs including *TaSNAC11-4B*-, *TaSNAC11-4B-N*-, and *TaSNAC11-4B-C*-pBridge and empty vector were transformed into the AH109 yeast strain and transformants were screened on SD-Trp medium for 3 days. Independent transformants were transferred to SD-Trp medium plates supplied with α-X-gal (20 mg·L^−1^) and grown for 3 days. Meanwhile, X-gal activity was measured by liquid assay using CPRG as substrate according to the protocol of the ProQuest^TM^ Two-Hybrid System with Gateway Technology from Invitrogen.

### 4.8. Dual-Luciferase Assay

The promoter regions of *Atrboh* genes were cloned using primers *AtrbohD*-pGreen-F/R and *AtrbohF*-pGreen-F/R (Table S1) and introduced into the pGreenII0800-LUC reporter vector. The sequence-confirmed reporter constructs were co-transformed with *35S*::*TaSNAC11-4B* into the Agrobacterium strain GV3101 which was used in transient transformation of *Nicotiana benthamiana*. The dual-LUC assay was performed with the Dual-Luciferase Reporter Assay System (Promega, WI, USA) on an enzyme labeling instrument (TECAN, Infinite M200 PRO, Männedorf, Switzerland). The LUC/REN ratio reflects the regulation of the *Atrboh* genes by TF *TaSNAC11-4B*.

## Supplementary Materials

Supplementary materials are available online at https://www.mdpi.com/1422-0067/21/20/7672/s1.

## Abbreviations

ABA	Abscisic acid
ROS	Reactive oxygen species
GA	Gibberellins
NADPH	Nicotinamide adenine dinucleotide phosphate
MeJA	Methyl jasmonate
SA	Salicylic acid
IAA	Indole-3-acetic acid
