# Supplementary material for: Wheat Transcription Factor TaSNAC11-4B Positively Regulates Leaf Senescence through Promoting ROS Production in Transgenic Arabidopsis
Source: Int J Mol Sci. 2020 Oct 16;21(20):7672. doi: 10.3390/ijms21207672 (PMC7589474; doi:10.3390/ijms21207672)
Supplement: Supplementary file 1 [file ijms-21-07672-s001.pdf]

Supplementary Figures

A

Percent Identity

Divergence	1	2	3	4	5	6	7	8		
1		97.4	94.3	58.3	58.9	59.2	74.4	51.3	1	TaSNAC11-4A
2	2.6		95.1	58.1	58.7	58.7	74.1	51.7	2	TaSNAC11-4B
3	5.9	5.1		57.6	57.9	57.9	73.8	50.6	3	TaSNAC11-4D
4	60.0	60.5	61.5		94.4	95.5	56.6	49.3	4	TaSNAC13-2A
5	58.8	59.2	60.9	5.8		97.7	57.8	48.9	5	TaSNAC13-2B
6	58.2	59.2	60.9	4.7	2.3		57.8	48.9	6	TaSNAC13-2D
7	31.4	31.9	32.3	63.8	61.2	61.2		51.3	7	OsNAP
8	76.4	75.4	78.4	81.9	82.9	82.9	76.4		8	AtNAP
	1	2	3	4	5	6	7	8		

B

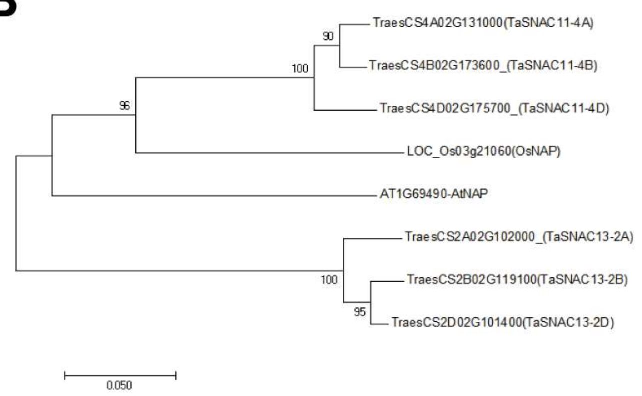

Supplementary Figure 1 Protein sequence alignment and phylogeny of TaSNAC 11-4B with other NAC homologues including OsNAP and AtNAP. (**A**) Identity and divergence of analyze among TaSNAC11-4B and other NAC proteins as indicated using MegAlign software. (**B**) Neighbor-joining (NJ) phylogenetic tree among TaSNAC11-4B and other NAC proteins including OsNAP and AtNAP.

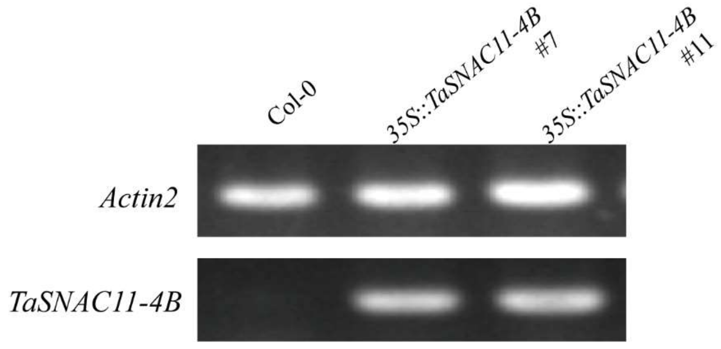

Supplementary Figure 2 Expression of *TaSNAC11-4B* in different lines as indicated.

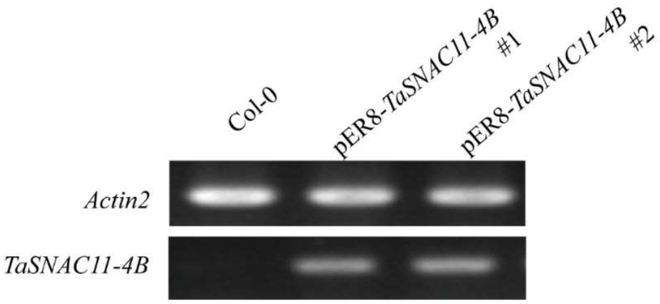

Supplementary Figure 3 Expression of *TaSNAC11-4B* in Col-0 and EST-inducible transgenic plants.

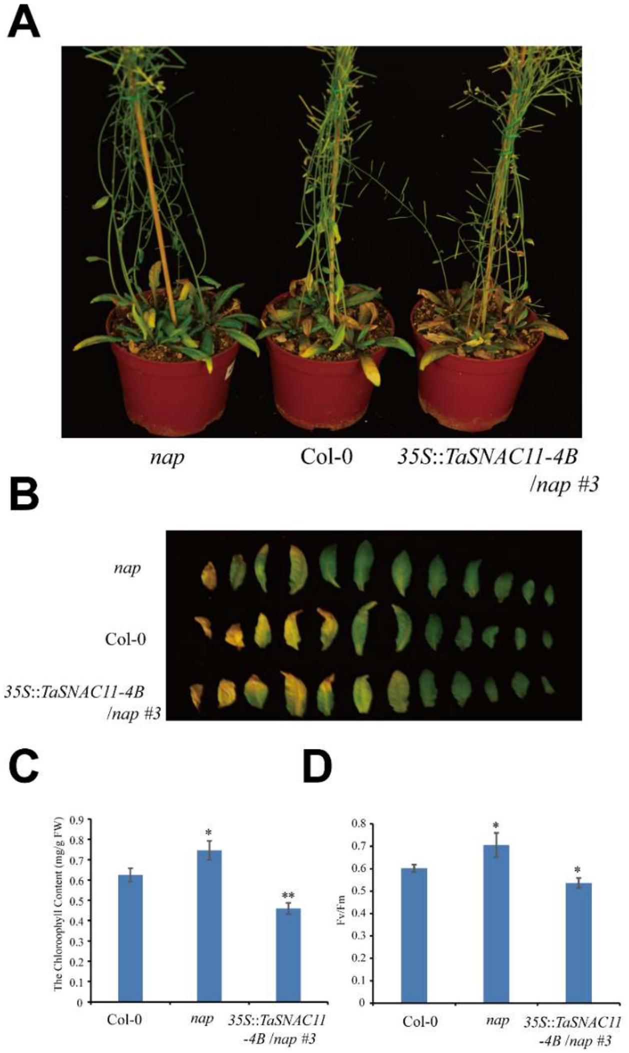

Supplementary Figure 4 Phenotype analysis of *35S*::*TaSNAC11-4B*/*nap* #3.(**A**) Phenotypes of whole plants of *nap* null mutant, Col-0 and *35S*::*TaSNAC11-4B*/*nap* #3. (**B**) Phenotypes of individual rosette leaves of *nap* null mutant,Col-0 and *35S*::*TaSNAC11-4B*/*nap* #3. (**C–D**) Chlorophyll content and Fv/Fm ratio of the sixth leaf from plants of different genotypes. Asterisks indicate statistically significant differences (* p <0.05; ** p <0.01) using student's *t*-test. Three independent experiments were performed.

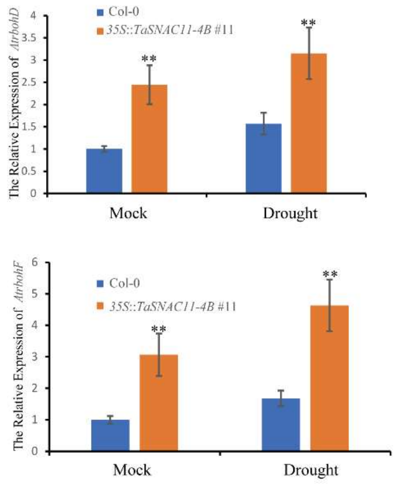

Supplementary Figure 5. Expression analysis of *AtrbohD* and *AtrbohF* genes in Col-0 and TaSNAC11-4B #11 overexpression plants.

Supplementary Table 1. Primers used in this study.

Primers Name	Primers Sequences (5'-3')
Q_SAG12_F	TCCAATTCTATTCGTCTGGTGTGT
Q_SAG12_R	CCACTTTCTCCCCATTTTGTTC
Q_ACT2_F	TGTGCCAATCTACGAGGGTTT
Q_ACT2_R	TTTCCCGCTCTGCTGTTGT
Q_RBCS3B_F	AGTAATGGCTTCCTCTATGC
Q_RBCS3B_R	GTGATGTCCTTGTTGGTCTTG
Q_SAG13_F	TGTCTGCGACGTCTCTTCT
Q_SAG13_R	TTGAATATTGACGTTCCCA
TaSNAC11-4B-QF	ACAGCATGCCGCAGCTTGGG
TaSNAC11-4B-QR	CTGGCTGGCTCGTCGACCCG
TaGAPDH-QF	TTAGACTTGCGAAGCCAGCA
TaGAPDH-QR	AAATGCCCTTGAGGTTTCCC
pBridgeTaSNAC11-4BF	TGTATCGCCGGAATTCATGATCATGTCCGACCCGGC
pBridge TaSNAC11-4BR	GCAGGTCGACGGATCCTTAGAAACGGAGCAACGTCC
pBridge TaSNAC11-4B-NR	GCAGGTCGACGGATCCGAGGCGGTACTCGTGCATGA

pBridge TaSNAC11-4B-CF	TGTATCGCCGGAATTCATGGCTGCCGCCGACGCACACG
TaSNAC11-4B-OEF	GTGGGGACAAGTTTGTACAAAAAAGCAGGCTTCATGATCA TGTCCGACCCGGC
TaSNAC11-4B-OER	GTGGGGACCACTTTGTACAAGAAAGCTGGGTCTTAGAAAC GGAGCAACGTCC
TaSNAC11-4B-RTF	ATGATCATGTCCGACCCGGC
TaSNAC11-4B-RTR	GAGGCGGTACTCGTGCATGA
AtrbohD-qF	AGCTTCACAATTATTGCACGAG
AtrbohD-qR	TCTCCAGTTAGGTTTAGCGAAG
AtrbohF-qF	TATTGGAGACCATCTTGCTTGT
AtrbohF-qR	CGTTAAAACCGGTTAGTCGATC
AtrbohD -pGreen-F	CGGTATCGATAAGCTTGTCTCCATCTATGTATCTAT
AtrbohD -pGreen-R	TAGAACTAGTGGATCCCGAATTCGAGAAACCAAAAAGAT C
AtrbohF -pGreen-F	CGGTATCGATAAGCTTGTAGTCTACATCTAACCCATTTG
AtrbohF -pGreen-R	TAGAACTAGTGGATCCAGATCCAAAGTCGGAATTC
